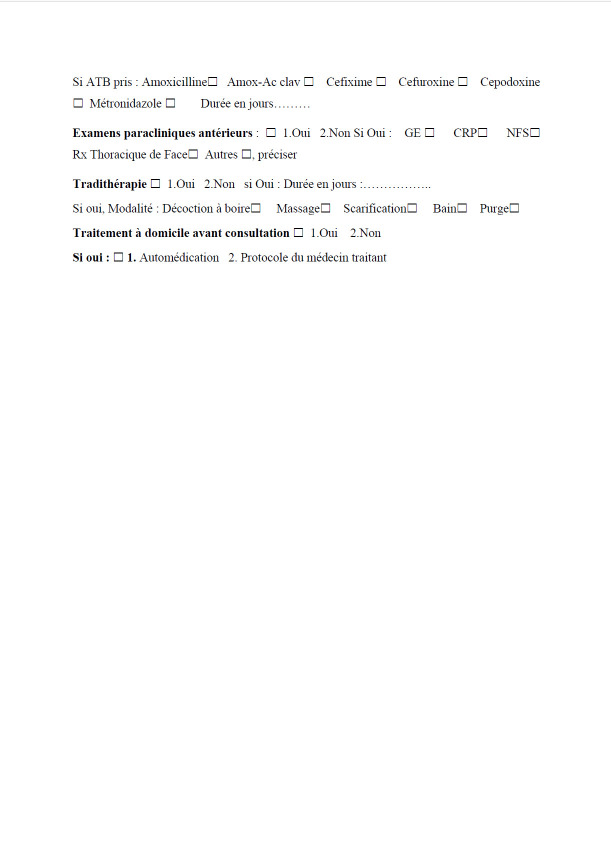# Parcours de soins des enfants drépanocytaires admis aux urgences pédiatriques du Centre hospitalier universitaire de Cocody (Abidjan, Côte d’Ivoire) de janvier à décembre 2024

**DOI:** 10.48327/mtsi.v6i2.2026.739

**Published:** 2026-05-04

**Authors:** André Marius GRO BI, Komenan Amoro MANSOU, Augustine DJIVOHESSOUN, Api Isabelle DJOMAN, Assai Prisca N’GATTA, Domé Charlène SORHO, Ahou Corine GOLI, Amorissani Amah FOLQUET

**Affiliations:** 1 Université Félix Houphouët Boigny de Cocody, Abidjan 01 BP V 34, Côte d’Ivoire; 2 Service de pédiatrie médicale, CHU de Cocody, Abidjan BP 22 V13, Côte d’Ivoire

**Keywords:** Drépanocytose, Parcours de soins, Automédication, Tradithérapie, Abidjan, Côte d’Ivoire, Afrique subsaharienne, Sickle cell disease, Care pathway, Self-medication, Traditional medicine, Abidjan, Côte d’Ivoire, Sub-Saharan Africa

## Abstract

**Introduction:**

La drépanocytose est pourvoyeuse de nombreuses complications nécessitant un recours fréquent aux soins. L’objectif de ce travail était d’analyser le parcours de soins des enfants atteints de syndrome drépanocytaire majeur admis aux urgences pédiatriques du Centre hospitalier universitaire (CHU) de Cocody, en vue d’identifier les facteurs associés au retard de consultation et, par conséquent, de favoriser une prise en charge précoce et adaptée des complications.

**Méthodologie:**

Il s’agissait d’une étude rétrospective à visée descriptive réalisée au service de pédiatrie du CHU de Cocody du 1er janvier au 31 décembre 2024 soit sur une durée de 12 mois. Les enfants porteurs d’un syndrome drépanocytaire majeur, avec un profil électrophorétique connu et hospitalisés pour une complication aiguë de la maladie ont été inclus. Le parcours de soins a été analysé. Les données ont été saisies et analysées à l’aide du logiciel Excel. Le test exact de Fisher a été utilisé pour comparer les proportions avec un seuil de signification de 5 % (p< 0,05).

**Résultats:**

Sur 800 admissions, 60 enfants présentant un syndrome drépanocytaire majeur ont été inclus, soit une prévalence hospitalière de 7,5 %. L’âge moyen était de 5,38 ans ± 5,3 avec des extrêmes de 6 mois et 14 ans. Le sex-ratio était de 1,07. La moitié des patients (50 %) a été diagnostiquée avant l’âge de 2 ans, souvent à l’occasion des manifestations cliniques telles que l’anémie (35 %), les crises vaso-occlusives (18,3 %) et le syndrome pied-main (16,7 %). La moyenne d’âge de découverte était de 1,8 an ± 8,9 avec des extrêmes de 3 mois et 12 ans. Le suivi était mauvais dans 61,7 % des cas. Dans 68,3 % des cas, la vaccination était incomplète ou non documentée. Les patients étaient référés d’un centre de santé dans 50 % des cas. Les patients ayant reçu un traitement avant référence représentaient 73,3 % des cas. Les antalgiques/antipyrétiques, les antipaludiques et les antibiotiques étaient les classes thérapeutiques les plus prescrites en ambulatoire avec respectivement 63,3 %, 23,3 % et 60 % des cas. Un retard à la consultation a été observé dans 71,7 % des cas, lié à un niveau socio-économique défavorable (p = 0,036), l’automédication (p = 0,023), le recours à la tradithérapie (p = 0,02), la mauvaise qualité du suivi (p = < 10-3) et la prise d’antalgique à domicile (p = < 10-3).

**Conclusion:**

Cette étude met en évidence plusieurs insuffisances dans le parcours de soins des enfants drépanocytaires, notamment le retard à la consultation lié à des facteurs socio-économiques et culturels. Une prise en charge plus structurée et éducative est indispensable pour améliorer leur pronostic.

**Figure:**
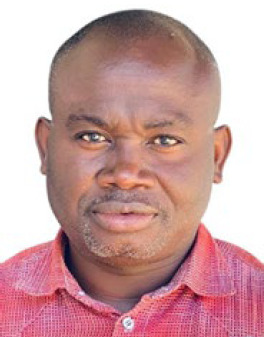

## Abstract (en)

**Introduction:**

Sickle cell disease leads to numerous complications that require frequent medical care. This study aimed to analyze the care pathway of children with severe sickle cell syndrome who were admitted to the pediatric emergency department at Cocody University Hospital. The goal was to identify factors associated with delayed consultation and promote the early and appropriate management of complications.

**Methodology:**

This retrospective descriptive study was conducted in the pediatrics department at Cocody University Hospital from January 1 to December 31, 2024. Children with major sickle cell syndrome and a known electrophoretic profile who were hospitalized for an acute complication of the disease were included in the study. The care pathway was analyzed. Data were entered and analyzed using Excel software. Fisher’s exact test was used to compare proportions, setting the significance threshold at 5% (p < 0.05).

**Results:**

Of the 800 admissions, 60 children with major sickle cell disease were included, representing a hospital prevalence of 7.5%. The mean age was 5.38 years, ranging from six months to 14 years. The sex ratio was 1.07. Fifty percent of the patients were diagnosed before the age of two, often due to clinical manifestations such as anemia (35%), vaso-occlusive crises (18.3%), and hand-foot syndrome (16.7%). The mean age at diagnosis was 1.8 years ± 8.9, ranging from three months to 12 years. Follow-up was inadequate in 61.7% of cases. In 68.3% of cases, vaccination status was incomplete or undocumented. Patients were referred from a health center in 50% of cases. Of those who received treatment prior to referral, 73.3% were included in the study. The most commonly prescribed drug classes in the outpatient setting were analgesics/antipyretics, antimalarials, and antibiotics, accounting for 63.3%, 23.3%, and 60% of cases, respectively. Delayed consultation was observed in 71.7% of cases and was associated with unfavorable socioeconomic status (p = 0.036), self-medication (p = 0.023), use of traditional medicine (p = 0.02), poor follow-up quality (p < 10^-3^), and taking analgesics at home (p < 10^-3^).

**Conclusion:**

This study highlights several shortcomings in the care pathway for children with sickle cell disease, particularly delays in seeking medical consultation linked to socioeconomic and cultural factors. A more structured, educational approach to care is essential to improving their prognosis.

## Introduction

La drépanocytose est une affection génétique héréditaire majeure caractérisée par une anomalie de l’hémoglobine, conduisant à une anémie chronique, des crises vaso-occlusives douloureuses et de multiples complications aiguës ou chroniques [4]. Elle touche particulièrement les populations d’Afrique subsaharienne, où sa prévalence est élevée [12]. En Côte d’Ivoire, la drépanocytose constitue un véritable problème de santé publique avec une prévalence du trait drépanocytaire estimée à environ 12 à 14 % [1]. La morbidité et la mortalité infantile sont encore préoccupantes, en dépit des efforts de sensibilisation et des protocoles de prise en charge [13].

Le service des urgences pédiatriques du Centre hospitalier universitaire (CHU) de Cocody reçoit chaque année de nombreux enfants drépanocytaires, souvent dans des contextes cliniques graves [6]. Ces admissions répétées posent la question de l’efficacité du parcours de soins en amont : quelles structures ont été consultées avant l’arrivée à l’hôpital ? Quels soins ont été prodigués ? Quelles barrières sociales et économiques ont influencé le recours aux soins ? Autant de questions essentielles pour comprendre les déterminants d’une prise en charge optimale.

Dans un contexte où la continuité des soins et la détection précoce des complications pourraient améliorer considérablement le pronostic de la maladie, il devient essentiel d’analyser le parcours de soins des enfants drépanocytaires avant leur admission aux urgences. Une telle analyse permettra de mieux orienter les stratégies d’intervention pour une prise en charge globale et efficiente de la drépanocytose pédiatrique.

Cette étude visait à analyser le parcours de soins des enfants porteurs de syndrome drépanocytaire majeur admis aux urgences pédiatriques du CHU de Cocody, pour identifier les facteurs associés au retard de consultation et, par conséquent, favoriser une prise en charge précoce et adaptée des complications.

## Matériel et méthodes

Notre travail s’est déroulé au Pôle gynéco-obstétrique et pédiatrique (PGOP), service de pédiatrie médicale du CHU de Cocody. Il s’agissait d’une étude rétrospective à visée descriptive et analytique couvrant la période du 1^er^ janvier au 31 décembre 2024, soit sur une durée de 12 mois.

La population d’étude était constituée de tous les enfants drépanocytaires admis aux urgences pédiatriques du PGOP du CHU de Cocody durant la période d’étude. Tous les enfants atteints de syndrome drépanocytaire majeur, exclusivement de génotype homozygote SS à l’électrophorèse de l’hémoglobine et admis aux urgences de pédiatrie pour prise en charge d’une complication aigüe liée à la maladie ont été inclus. Les patients ayant un dossier inexploitable (incomplet ou introuvable) avec un profil électrophorétique non disponible n’ont pas été inclus dans l’étude. Il s’agissait d’un échantillonnage de type exhaustif. Les variables étudiées étaient les données socio-démographiques (âge, sexe, niveau d’instruction, provenance, niveau socio-économique des parents), les données anamnestiques et de suivi (circonstances de découverte de la maladie, âge de découverte, antécédents vaccinaux, qualité du suivi, observance thérapeutique) et les données sur le parcours de soins avant admission aux urgences (lieu de consultation, délai entre l’apparition des signes et l’arrivée à l’hôpital, traitements administrés avant admission, recours à la médecine traditionnelle, automédication).

Les données ont été recueillies sur une fiche d’enquête individuelle à partir des dossiers médicaux (voir Annexe). La collecte des données a été réalisée par les médecins en spécialité de pédiatrie sous la supervision de l’équipe encadrante. Leur saisie et leur analyse ont été faites à l’aide du logiciel Excel. Pour les variables qualitatives, nous avons utilisé le calcul des proportions. Pour les variables quantitatives, nous avons calculé les moyennes, les écarts types et les extrêmes. Le niveau socioéconomique de la famille a été apprécié selon la classification de Gayral-Taminh *et al.* [5]. Il s’agit d’un outil simple d’évaluation du niveau socioéconomique familial, élaboré dans le contexte ivoirien des années 1980 mais encore largement utilisé en Afrique de l’Ouest. Cette classification attribue à chaque famille un niveau (élevé, moyen ou faible) selon la majorité des critères observés (Tableau I), le niveau le plus bas étant retenu en cas d’égalité. Le suivi était jugé bon lorsque l’enfant totalisait au moins quatre consultations par an avec une réalisation trimestrielle de la numération formule sanguine, la créatininémie, et la réalisation annuelle de la ferritinémie, d’une échographie abdominale et cardiaque et d’un bilan ophtalmologique. L’observance thérapeutique était jugée bonne lorsque la prophylaxie par l’acide folique et le Tanakan ou l’hydroxyurée était régulière. L’évaluation de l’observance à la pénicilline V n’a pas été faite car sa disponibilité demeure irrégulière dans notre contexte. L’état vaccinal était bon lorsque le sujet était à jour sur les vaccins du programme élargi de vaccination en Côte d’Ivoire (DTCP et hépatite virale B) et les vaccins recommandés aux sujets atteints de drépanocytose (Typhim Vi, ROR, Pneumo 23, vaccin méningococcique ACWY). La médecine traditionnelle désignait diverses pratiques, approches, connaissances et croyances sanitaires intégrant des médicaments à base de plantes, d’animaux et/ou de minéraux, des traitements spirituels, des techniques manuelles et exercices, appliqués seuls ou en association afin de maintenir le bien-être et traiter, diagnostiquer ou prévenir la maladie [10]. L’automédication désignait le comportement par lequel un patient recourt de sa propre initiative à un médicament, c’est-à-dire à une substance dont il attend un effet de type pharmacologique bénéfique pour sa santé [2]. Le retard à la consultation correspondait à toute consultation faite au-delà de 24 h d’évolution des signes. Le principal critère de jugement était l’existence de retard à la consultation et ses facteurs associés. Le test exact de Fisher a été utilisé pour comparer les proportions avec un seuil de signification de 5 % (p< 0,05). La taille restreinte de l’échantillon (n = 60) ne permettait pas de procéder à des analyses multivariées avec une puissance et robustesse suffisantes.

**Tableau I:** Critères de classification du niveau socio-économique selon Gayral-Taminh *et al*. [5]

Critères	Niveau élevé	Niveau moyen	Niveau faible
**Profession du chef de famille**	Cadres supérieurs, professions libérales, commerçants prospères	Employés moyens, petits commerçants, artisans qualifiés	Ouvriers non qualifiés, cultivateurs de subsistance, sans emploi
**Niveau d'instruction**	Études supérieures ou secondaires complètes	Secondaire partiel ou primaire complet	Analphabétisme ou primaire non achevé
**Revenus**	Stables et élevés, permettant un confort matériel	Revenus modestes mais réguliers	Revenus faibles, irréguliers ou inexistants
**Logement - Habitat**	Maison en dur bien équipée, accès à eau courante et électricité	Logement en semi-dur, parfois sans todas les commodités	Habitat précaire, souvent sans eau ni électricité
**Équipements ménagers**	Réfrigérateur, télévision, véhicule, biens durables multiples	Quelques biens (radio, ventilateur, vélo, téléviseur)	Très peu ou aucun équipement

Notre étude a reçu l’accord des différents responsables du CHU de Cocody. Elle a été faite sur des données du dossier médical, n’a pas eu de risque direct sur les participants et n’a pas nécessité de consentement écrit ou verbal. L’anonymat et la confidentialité des informations ont été strictement respectés.

## Résultats

Durant la période d’étude, 800 patients ont été admis aux urgences pédiatriques, parmi lesquels 63 (8 %) étaient atteints de syndrome drépanocytaire majeur. Après application des critères d’inclusion, 60 dossiers complets ont été retenus, soit une prévalence hospitalière de 7,5 %. Trois dossiers (4,7 %) ont été exclus car non exploitables. La tranche d’âge de 6 à 10 ans était la plus représentée avec 47 %. L’âge moyen était de 5,38 ans ± 5,3 avec des extrêmes allant de 6 mois à 14 ans. Le sex-ratio était de 1,07. Le niveau socio-économique était majoritairement faible dans 63 %, moyen dans 30 % et élevé dans 7 % des cas. Dans 93 % des cas, les enfants résidaient dans la ville d’Abidjan. La commune de Yopougon représentait 32 % des cas parmi les patients résidant dans la ville d’Abidjan, suivie de la commune d’Abobo dans 29 % des cas.

La majorité des enfants atteints de syndrome drépanocytaire majeur étaient diagnostiqués avant l’âge de deux ans. L’anémie représentait la principale circonstance de découverte, tandis que d’autres situations cliniques comme les douleurs ostéo-articulaires, le syndrome pied-main ou encore une découverte fortuite en hospitalisation contribuaient également au dépistage. Le suivi des patients apparaissait globalement insuffisant. Si le traitement de fond était prescrit dans la majorité des cas, la réalisation des bilans systématiques et l’application des protocoles de prise en charge à domicile restaient limitées. La couverture vaccinale était, quant à elle, souvent incomplète ou mal documentée. Les données relatives à la drépanocytose sont présentées dans le Tableau II. La durée d’évolution des signes avant le 1^er^ contact avec un centre de santé était supérieure à 24 h chez 43 patients, soit 72 % des cas. Les patients étaient référés d’un centre de santé dans 50 % des cas. Les patients ayant reçu un traitement avant référence représentaient 73 % des cas. La majorité des patients avaient eu recours à un traitement avant leur admission aux urgences. L’automédication constituait l’attitude la plus fréquente à domicile, suivie du recours au protocole prescrit par un médecin. La tradithérapie restait présente chez une proportion non négligeable de patients. Une grande partie des enfants avait déjà consulté avant leur admission, souvent à plusieurs reprises, avec une prise en charge essentiellement ambulatoire. Les examens paracliniques étaient réalisés dans un peu plus de la moitié des cas. Enfin, les prescriptions étaient dominées par les antalgiques/antipyrétiques et les antibiotiques. Le Tableau III résume l’attitude thérapeutique avant admission aux urgences pédiatriques du CHU de Cocody.

**Tableau II:** Répartition des patients selon les données relatives à la drépanocytose

Données relatives à la drépanocytose	Effectif	Pourcentage
Tranche d'âge de découverte de la maladie		
< 2 ans	30	50
2 - 5 ans	17	28
5 - 10 ans	7	12
10 - 15 ans	6	10,0
Circonstances de découverte de la maladie		
anémie	21	35
douleur ostéo-articulaire	11	18
syndrome pied-mains	10	17
fortuite (en hospitalisation)	9	15
douleur abdominale	7	12
bilan systématique	2	3
Qualité du suivi		
bonne	23	38
mauvaise	37	62
Contenu du suivi		
traitement de fond	48	80
bilan systématique annuel	12	20
protocole de prise en charge de la douleur à domicile	4	7
vaccination incomplète ou non documentée	41	68

**Tableau III:** Répartition des patients selon l’attitude thérapeutique avant admission aux urgences

Attitude thérapeutique avant admission aux urgences	Effectif	Pourcentage
Traitement à domicile (n = 60)		
oui	48	80
non	12	20
Type de traitement à domicile (n = 48)		
automédication	36	75
protocole du médecin traitant	12	25
Tradithérapie (n = 60)	
oui	17	28
non	43	72
Consultation avant admission (n = 60)		
oui	43	72
non	17	28
Nombre de consultations effectuées (n = 43)		
une consultation	26	61
au moins deux consultations	17	39
Traitement ambulatoire pendant la première consultation (n = 43)		
oui	32	74
non	11	25
Examens paracliniques au cours de la première consultation (n = 43)		
oui	26	61
non	17	39
Principaux traitements reçus (n = 60)		
antalgiques/antipyrétiques	41	68
antibiotiques	36	60
antipaludiques	14	23

Plusieurs facteurs étaient significativement associés au retard de consultation. Les plus déterminants étaient les conditions socio-économiques défavorables et la mauvaise qualité du suivi, qui augmentaient fortement le risque de retard. L’utilisation d’antalgiques à domicile, l’automédication et le recours à la tradithérapie contribuaient également à prolonger le délai d’accès aux soins. Les facteurs associés au retard à la consultation sont résumés dans le Tableau IV.

**Tableau IV:** Répartition des patients selon les facteurs associés au retard à la consultation

	Retard à la consultation		OR [IC à 95 %]
	Oui	Non	
Condition socio-économique			
élevée ou moyenne	6 (27 %)	16 (73 %)	0,0115 [0,0002; 0,0961]
faible	37 (97 %)	1 (3 %)	
Antalgique à domicile			
oui	40 (83 %)	8 (17 %)	14,0534 [2,7646; 99,0895]
non	3 (25 %)	9 (75 %)	
Qualité du suivi			
bonne	8 (35 %)	15 (65 %)	0,0331 [0,0031; 0,1824]
mauvaise	35 (95 %)	2 (5 %)	
Automédication			
oui	30 (83 %)	6 (17 %)	4,1195 [1,1165; 16,8024]
non	13 (54 %)	11 (45 %)	
Tradithérapie			
oui	16 (94 %)	1 (6 %)	9,2174 [ 1,2012; 421,1291]
non	27 (63 %)	16 (37 %)	

## Discussion

Le service de pédiatrie du CHU de Cocody a mis en place, depuis près de trois ans, une cohorte de suivi des enfants drépanocytaires. Celle-ci regroupe des patients diagnostiqués en périphérie, puis orientés vers le service pour leur suivi, ainsi que des enfants identifiés au cours d’une hospitalisation pour complication, et intégrés à la cohorte après leur prise en charge. La prise en charge des patients drépanocytaires dans notre service reste peu structurée. Des associations de lutte contre la drépanocytose interviennent principalement dans la sensibilisation et apportent un appui à l’éducation thérapeutique des patients. Toutefois, l’ensemble des coûts liés aux soins, incluant les médicaments, les examens biologiques, l’hospitalisation et la vaccination demeure entièrement à la charge des parents.

La fréquence des complications aiguës de la drépanocytose parmi les admissions aux urgences était élevée. Ce taux élevé observé dans notre étude pourrait s’expliquer par la fermeture du CHU de Yopougon, qui abritait le service d’hématologie clinique, centre de référence pour la prise en charge de la drépanocytose. Cette fermeture a conduit à une redirection des patients vers le CHU de Cocody.

Le niveau socio-économique était majoritairement faible. Cette situation pourrait être un frein au suivi correct des patients drépanocytaires. Une association statistiquement significative a été retrouvée entre les conditions socio-économiques et le délai de consultation (p < 10^-3^). Les patients issus de milieux défavorisés consultaient plus tardivement, probablement en raison de revenus insuffisants limitant l’accès rapide aux soins. Ces résultats suggèrent que les moyens financiers constituent un déterminant majeur dans le parcours de soins des drépanocytaires.

La découverte du statut drépanocytaire a été faite souvent à l’occasion des manifestations cliniques telles que l’anémie, les crises vaso-occlusives à localisation ostéoarticulaire ou abdominale chez les grands enfants et le syndrome pied-main chez les nourrissons. Cette situation est fréquemment observée dans les pays à ressources limitées. À titre d’exemple, en Ouganda, en 2018, Hernandez *et al*. ont rapporté des prévalences de crises vasoocclusives et de syndrome pied-main de 48 % et 25 % respectivement [7]. Ce constat pourait s’expliquer par une absence de dépistage néonatal de la drépanocytose. Les complications constituent ainsi la circonstance de découverte dans notre contexte où le dépistage systématique de la drépanocytose était rarement fait. Par contre, en Europe et précisément en France, le dépistage se fait de façon systématique en période néonatale [3]. La découverte tardive met aussi en évidence les insuffisances de notre système de santé. En cas d’anémies répétées ou de douleurs abdominales chez l’enfant, le réflexe du praticien devrait être de prescrire une électrophorèse de l’hémoglobine en tant qu’examen de première intention, ce qui n’est pas systématiquement fait.

Le suivi des patients apparaissait globalement insuffisant. Dans notre contexte, la prise en charge des patients drépanocytaires n’est pas couverte par un système d’assurance maladie universel. Bien que certaines organisations non gouvernementales apportent un soutien ponctuel à quelques familles, ces aides demeurent limitées et ne concernent pas l’ensemble du territoire national. Parallèlement, les associations de patients atteints de drépanocytose jouent un rôle essentiel dans l’amélioration de la prise en charge globale de cette affection chronique. Généralement constituées sous forme d’organisations à but non lucratif, ces associations sont souvent initiées et animées par des patients euxmêmes, leurs parents ou des professionnels de santé. Elles interviennent à plusieurs niveaux, notamment par le soutien psychosocial aux familles, l’éducation thérapeutique des patients, ainsi que la sensibilisation du grand public afin de favoriser le dépistage précoce et de réduire la stigmatisation associée à la maladie.

En outre, ces structures participent activement au plaidoyer auprès des autorités sanitaires pour une meilleure reconnaissance de la drépanocytose comme problème majeur de santé publique. Elles contribuent également à l’organisation de campagnes de dépistage, à la formation des patients et des aidants, et offrent parfois une aide financière ou matérielle pour l’accès aux soins (médicaments, examens complémentaires ou hospitalisations). Cependant, malgré leur engagement, l’impact de ces associations reste limité par plusieurs contraintes, notamment l’insuffisance de ressources financières, la dépendance vis-à-vis de partenaires extérieurs, le manque de structuration organisationnelle et une couverture géographique restreinte. Le renforcement de leurs capacités institutionnelles et leur intégration dans les programmes nationaux de santé apparaissent donc essentiels pour améliorer durablement la qualité de vie et le suivi médical des patients drépanocytaires.

Les frais liés aux examens biologiques, aux hospitalisations et aux médicaments essentiels tels que l’hydroxyurée ou les antibiotiques restent majoritairement à la charge des familles. Cette réalité socio-économique fragilise l’accès aux soins, encourage le recours à l’automédication et à la tradithérapie, et pourrait expliquer un suivi médical irrégulier chez de nombreux patients. Toutefois, le recours à la tradithérapie ne saurait être expliqué uniquement par ces contraintes financières. Il s’inscrit également dans des logiques socioculturelles et des comportements de recours aux soins, où les choix thérapeutiques des familles sont influencés par les croyances.

Un autre point important mis en évidence par cette étude est l’absence de structuration du parcours de soins pour les enfants atteints de drépanocytose. Actuellement, il n’existe pas de circuit formalisé de prise en charge, ce qui fragilise la continuité et la coordination des soins. Cette désorganisation entraîne une gestion fragmentée, avec des ruptures dans le suivi médical, un recours irrégulier aux consultations et une difficulté à assurer l’observance thérapeutique sur le long terme. La mise en place d’un centre de référence ou d’unités spécialisées dédiées à la drépanocytose permettrait d’organiser un parcours de soins cohérent, centré sur le patient, et d’améliorer à la fois la prévention des complications et la qualité de vie des patients.

La drépanocytose étant une pathologie chronique dont le traitement curatif n’est pas accessible actuellement, l’amélioration du pronostic passe par un suivi régulier afin de prévenir les complications pouvant mettre en jeu le pronostic vital [8]. Notre étude a mis en évidence une association significative entre la qualité du suivi médical et le délai de consultation (p < 10^-3^). Les enfants bénéficiant d’un suivi irrégulier présentaient plus fréquemment un retard à la consultation. À l’inverse, un suivi régulier et de bonne qualité apparaissait comme un facteur protecteur, facilitant une prise en charge plus précoce. Les données de la littérature soulignent l’importance d’un suivi structuré et continu dans l’anticipation des complications et l’orientation rapide vers les structures de soins en cas de crise [5].

La couverture vaccinale était très souvent incomplète ou mal documentée. Ce faible taux de couverture vaccinale pourrait s’expliquer par l’ignorance, la négligence et le manque de sensibilisation sur la nécessité de vacciner correctement les enfants drépanocytaires. Les vaccins hors Programme élargi de vaccination préconisés par l’Organisation mondiale de la santé pour les drépanocytaires ne sont pas accessibles dans notre contexte à cause de leur coût [11]. Les enfants drépanocytaires devraient bénéficier gratuitement de ces vaccins car étant très sensibles aux infections à germes encapsulés (pneumocoque, méningocoque, *Haemophilus*, salmonelle). L’absence d’une couverture vaccinale satisfaisante chez ces enfants drépanocytaires est source de complications infectieuses graves. Ce constat souligne l’importance de renforcer les stratégies de vaccination, d’améliorer l’éducation thérapeutique des familles et de promouvoir un suivi médical plus rigoureux.

Dans notre étude, une proportion importante d’enfants avait déjà consulté avant leur admission, parfois à plusieurs reprises. Ce phénomène peut s’expliquer par le fait que les parents se dirigent vers le centre de référence seulement lorsque les premiers soins administrés en périphérie se révèlent inefficaces. Cette tendance est confirmée par Olatunya *et al*. au Nigeria, qui ont rapporté un taux de première consultation de 58,2 %, mettant en évidence l’application progressive de la pyramide sanitaire [9].

Les antalgiques/antipyrétiques, les antipaludiques et les antibiotiques étaient les classes thérapeutiques les plus prescrites. Cette prédominence est commune à plusieurs pays africains tel que le Nigéria [9]. Cette situation serait probablement en rapport avec la diversité des complications observées chez les enfants porteurs de syndrome drépanocytaire majeur.

Les patients ont consulté au-delà de 24 heures dans 71,7 % des cas.

Dans notre étude, l’automédication, le recours à la tradithérapie et l’administration d’antalgiques à domicile étaient significativement associés au retard de consultation (respectivement, p = 0,023; p = 0,02; p < 10^-3^). Ces pratiques, souvent intriquées, peuvent masquer transitoirement les symptômes ou induire une perception d’amélioration, retardant ainsi le recours aux soins formels. Ce retard compromet la prise en charge précoce des complications et peut aggraver le pronostic. Des interventions ciblées sont nécessaires, combinant une amélioration de l’accessibilité financière et géographique aux soins, notamment par la mise en place de structures de proximité et le renforcement de l’éducation thérapeutique des familles. Celle-ci doit insister sur la reconnaissance précoce des signes de gravité et les limites de l’automédication. L’intégration des tradipraticiens dans des stratégies de collaboration pourrait également favoriser une orientation plus rapide vers les structures de santé.

Cette étude comporte certaines limites qu’il convient de souligner.

D’abord, sa réalisation rétrospective expose à des données incomplètes, notamment concernant certains éléments du suivi clinique et la profession des parents. Ensuite, la taille de l’échantillon est restreinte, ce qui limite la réalisation d’analyses multivariées. Par ailleurs, la population consultant au CHU de Cocody n’est probablement pas représentative de l’ensemble des enfants drépanocytaires du district d’Abidjan. Des contraintes financières peuvent freiner l’accès à ce centre, notamment pour les familles à faibles revenus, qui seraient alors sous-représentées dans notre échantillon. Ce biais de sélection doit être pris en compte dans l’interprétation et la généralisation des résultats. Enfin, l’absence de données exhaustives sur les coûts réellement supportés par les familles limite l’analyse économique fine du parcours de soins.

## Conclusion

L’évaluation du parcours de soins de l’enfant drépanocytaire montre des insuffisances : d’une part, le retard à la consultation, favorisé par des conditions socio-économiques défavorables, l’automédication, la tradithérapie et l’irrégularité du suivi et, d’autre part, l’absence d’un circuit clair et structuré de prise en charge. Ces limites compromettent la santé et le pronostic vital des enfants, soulignant l’urgence d’interventions adaptées. Le renforcement de l’accessibilité financière et géographique aux soins, associé à une éducation thérapeutique renforcée des familles, constitue une stratégie essentielle pour améliorer la compréhension de la maladie, encadrer l’automédication, assurer un suivi rigoureux et reconnaître précocement les signes de gravité. Par ailleurs, l’implication des tradipraticiens, dans une approche respectueuse du contexte socioculturel, devrait contribuer à réduire les retards de consultation. Enfin, la création d’un centre de référence dédié à la drépanocytose apparaît indispensable pour structurer et coordonner la prise en charge, garantir la continuité des soins et améliorer durablement le pronostic des enfants. Au regard des limites identifiées, il serait pertinent de réaliser des études multicentriques et prospectives sur de larges échantillons afin d’améliorer la représentativité des résultats, tout en mettant en place une collecte systématique des coûts liés au suivi des patients, incluant les consultations, les examens, les traitements et les hospitalisations.

## Sources de financement

Aucune source de financement externe n’a été reçue pour la réalisation de cette étude.

## Contributions des auteurs et autrices

GRO BI André Marius : conception de l’étude, collecte et analyse des données, rédaction du manuscrit.

MANSOU Komenan Amoro, DJIVOHESSOUN Augustine, ASSI-SAHOUIN Ursule Anièla, DJOMAN Isabelle, KOUADIO Evelyne, DAINGUY Marie Evelyne, KOUAKOU Cyprien, AKE-ASSI Marie Hélène, N’GATTA Prisca, SORHO Charlène : relecture critique, validation des résultats, révision du manuscrit.

FOLQUET Amorissani : validation du protocole, révision du manuscrit.

## Déclaration de liens d’intérêts

Aucun lien d’intérêt n’a été déclaré.

## Annexe : Fiche d’enquête individuelle

**Figure:**
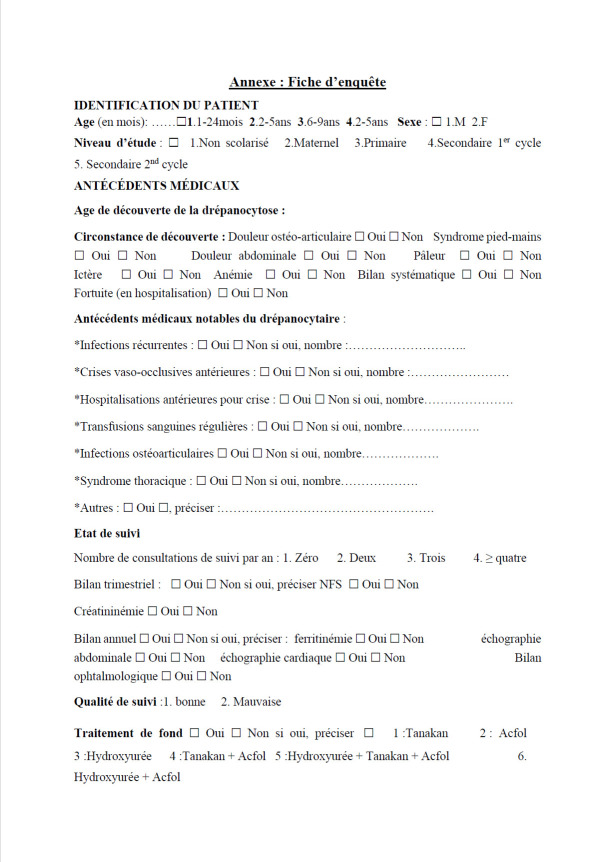

**Figure:**
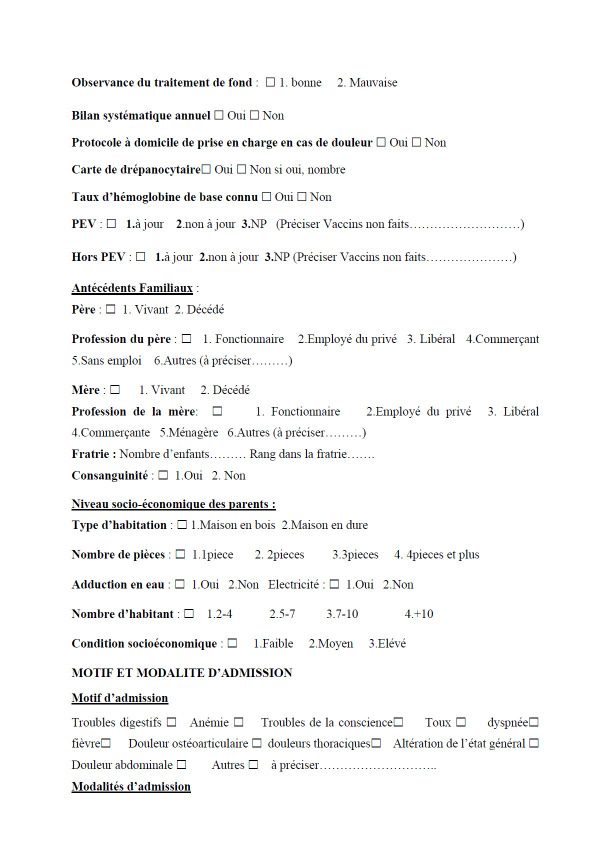

**Figure:**
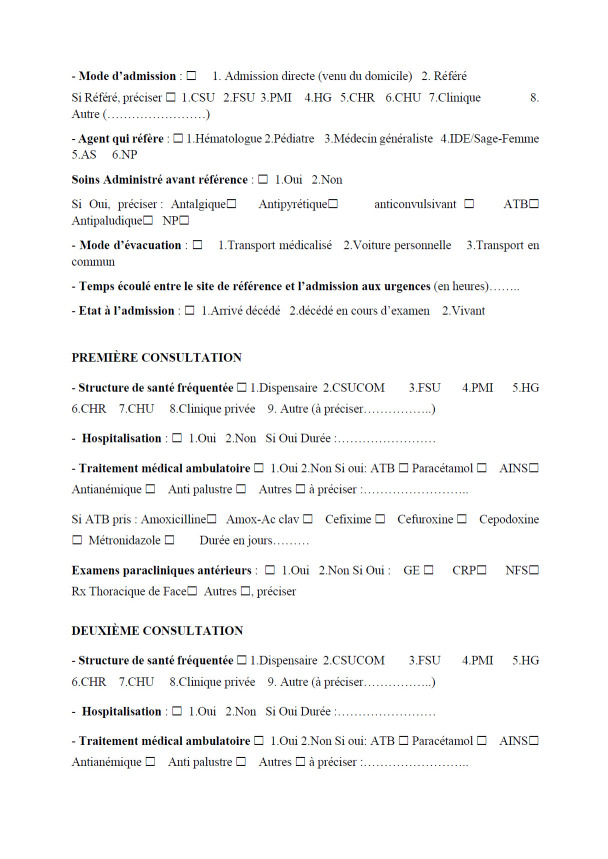

**Figure:**