# Supplementary material for: Care pathway for children with sickle cell disease admitted to the pediatric emergency department at the Cocody University Hospital (Abidjan, Ivory Coast) from January to December 2024
Source: Med Trop Sante Int. 2026 May 4;6(2):mtsi.v6i2.2026.739. [Article in French] doi: 10.48327/mtsi.v6i2.2026.739 (PMC13423302; doi:10.48327/mtsi.v6i2.2026.739)
Supplement: Related texts

# Research article: Care pathway for children with sickle cell disease admitted to the pediatric emergency department at the Cocody University Hospital (Abidjan, Ivory Coast) from January to December 2024

## Introduction

Sickle cell disease (SCD) is a major hereditary disorder involving a hemoglobin abnormality. It leads to chronic anemia and painful vaso-occlusive crises, as well as numerous acute and chronic complications [4]. The disease particularly affects populations in sub-Saharan Africa, where it is highly prevalent [12]. In Côte d’Ivoire, SCD is a significant public health concern, with an estimated 12–14% prevalence of the sickle cell trait [1]. Despite awareness-raising efforts and established management protocols, morbidity and infant mortality remain a cause for concern [13]. The pediatric emergency department at Cocody University Hospital admits numerous children with SCD each year, often in serious clinical condition [6]. These repeated admissions raise questions about the effectiveness of the care pathway: Which healthcare facilities were consulted before arriving at the hospital? What care was provided? What social and economic barriers influenced access to care? These are all essential questions for understanding the determinants of optimal care.

In a context where continuity of care and early detection of complications could significantly improve disease prognosis, analyzing the care pathway of children with SCD prior to their admission to the emergency department becomes essential. Such an analysis will help guide intervention strategies for the comprehensive and efficient management of pediatric SCD.

The aim of this study was to analyze the care pathway of children with major sickle cell syndrome who were admitted to the pediatric emergency department at Cocody University Hospital in order to identify factors associated with delayed consultation and to promote the early and appropriate management of complications.

## Materials and methods

Our study was conducted at the Gynecology-Obstetrics and Pediatrics Center (PGOP) at the Cocody University Hospital, in the medical pediatrics department. This retrospective study had descriptive and analytical objectives and covered the 12-month period from January 1 to December 31, 2024.

The study population consisted of all children with SCD who were admitted to the pediatric emergency department of the PGOP at the Cocody University Hospital during the study period. We included all children with major SCD of the SS homozygous genotype, as determined by hemoglobin electrophoresis, who were admitted to the pediatric emergency department for management of an acute complication related to the disease. Patients with unusable records (incomplete or missing) and an unavailable electrophoretic profile were excluded from the study. This was an exhaustive sample. The variables studied included sociodemographic data (age, sex, educational level, place of origin, and parents’ socioeconomic status); medical history and follow-up data (circumstances of disease discovery, age at discovery, vaccination history, quality of follow-up, and treatment adherence); and data on the care pathway prior to emergency department admission (place of consultation, time between symptom onset and arrival at the hospital, treatments administered before admission, use of traditional medicine, and self-medication).

Data were collected using individual survey forms based on medical records (see Appendix). Pediatric residents collected the data under the supervision of the supervising team. Data entry and analysis were conducted using Excel. For qualitative variables, proportions were calculated. For quantitative variables, we calculated means, standard deviations, and extremes. We assessed the family’s socioeconomic status according to the classification by Gayral-Taminh *et al.* [5]. This simple tool for assessing family socioeconomic status was developed in the Ivorian context of the 1980s and is still widely used in West Africa. The classification system assigns each family a level (high, medium, or low) based on the majority of the observed criteria (Table I). In cases of a tie, the lowest level is selected. Follow-up was considered adequate if the child had at least four visits per year with complete blood counts and serum creatinine tests quarterly, serum ferritin tests annually, and abdominal and cardiac ultrasounds and an ophthalmological examination annually.

**Table I:** Criteria for classifying socioeconomic status according to Gayral-Taminh *et al.* [5]

Criteria	High level	Medium level	Low level
**Occupation of head of household**	Senior executives, professionals, successful business owners	Mid-level employees, small business owners, skilled craftsmen	Unskilled laborers, subsistence farmers, unemployed
**Level of education**	Higher education or complete secondary education	Partial secondary education or complete primary education	Illiteracy or incomplete primary education
**Income**	Stable and high income, allowing for material comfort	Modest but regular income	Low, irregular, or no income
**Housing - Living Conditions**	Well-equipped brick house, access to running water and electricity	Semi-permanent housing, sometimes lacking all amenities	Precarious housing, often without water or electricity
**Household Appliances**	Refrigerator, television, vehicle, multiple durable goods	A few items (radio, fan, bicycle, television)	Very few or no appliances

We considered therapeutic adherence good when prophylaxis with folic acid and Tanakan or hydroxyurea was regular. Adherence to penicillin V was not assessed due to its irregular availability in our setting. Vaccination status was considered adequate if the subject was up to date on the Expanded Program on Immunization (EPI) vaccines (DTCP and hepatitis B) in Côte d’Ivoire and the vaccines recommended for individuals with SCD (Typhim Vi, MMR, Pneumo 23, and the ACWY meningococcal vaccine). Traditional medicine refers to various health practices, approaches, knowledge, and beliefs that incorporate herbal, animal, and/or mineral-based medicines; spiritual treatments; manual techniques; and exercises. These practices can be applied alone or in combination to maintain well-being, treat, diagnose, or prevent disease [10]. Self-medication refers to a patient’s use of a medication on their own initiative, expecting a beneficial pharmacological effect on their health [2]. Delay in seeking medical care was defined as any consultation occurring more than 24 hours after the onset of symptoms. The primary outcome measure was the presence of a delay in seeking medical care and its associated factors. Fisher’s exact test was used to compare proportions, setting a significance threshold of 5% (p < 0.05). Due to the small sample size (n = 60), multivariate analyses were not feasible.

Our study was approved by the relevant authorities at Cocody University Hospital. Based on medical record data, it posed no direct risk to participants and did not require written or verbal consent. The anonymity and confidentiality of the information were strictly maintained.

## Results

During the study period, 800 patients were admitted to the pediatric emergency department. Of those patients, 63 (8%) had major sickle cell syndrome. After applying the inclusion criteria, 60 complete records were selected, representing a hospital prevalence of 7.5%. Three cases (4.7%) were excluded because they were unusable.

The 6–10 age group was the most represented, accounting for 47% of the sample. The mean age was 5.38 years, with ages ranging from six months to 14 years. The sex ratio was 1.07. Socioeconomic status was low in 63% of cases, moderate in 30%, and high in 7%. Ninety-three percent of the children resided in the city of Abidjan. Among patients residing in Abidjan, the Yopougon district accounted for 32% of cases, followed by the Abobo district at 29%.

Most children with major sickle cell syndrome were diagnosed before the age of two. Anemia was the primary reason for diagnosis, while other clinical presentations, such as osteoarticular pain, hand-foot syndrome, and incidental discovery during hospitalization, also contributed to detection. Patient follow-up appeared generally inadequate. Although maintenance therapy was prescribed in most cases, routine check-ups and home care protocol implementation remained limited. Meanwhile, vaccination coverage was often incomplete or poorly documented. Data on SCD are presented in Table II.

**Table II:** Distribution of patients by sickle cell disease data

Data on sickle cell disease	Headcount	Percentage
Age at diagnosis			
< 2 years	30	50
2 - 5 years	17	28
5 -10 years	7	12
10 -15 years	6	10,0
Circumstances of diagnosis			
anemia	21	35
bone and joint pain	11	18
hand-foot syndrome	10	17
incidental (during hospitalization)	9	15
abdominal pain	7	12
routine check-up	2	3
Quality of follow-up			
good	23	38
poor	37	62
Content of follow-up			
maintenance therapy	48	80
annual routine check-up	12	20
home pain management protocol	4	7
incomplete or undocumented vaccination	41	68

The duration of symptoms prior to first contact with a health center exceeded 24 hours for 43 patients, accounting for 72% of cases. Fifty percent of patients were referred from a health center. Of those patients, 73% had received treatment prior to referral. Most patients had sought treatment before being admitted to the emergency department. Self-medication was the most common approach at home, followed by adherence to a physician-prescribed protocol. Traditional medicine remained prevalent among a significant proportion of patients. Many children had already consulted a healthcare provider before admission, often multiple times, with care primarily provided on an outpatient basis. Paraclinical tests were performed in just over half of the cases. Finally, prescriptions were dominated by analgesics, antipyretics, and antibiotics. Table III summarizes the therapeutic approach prior to admission to the pediatric emergency department at Cocody University Hospital.

**Table III:** Distribution of patients by treatment approach prior to admission to the emergency department

Therapeutic approach prior to emergency department admission	Headcount	Percentage
Home treatment (n = 60)			
yes	48	80
no	12	20
Type of home treatment (n = 48)			
self-medication	36	75
prescription from the primary care physician	12	25
Traditional therapy (n = 60)			
yes	17	28
no	43	72
Consultation prior to admission (n = 60)			
yes	43	72
no	17	28
Number of consultations (n = 43)			
one consultation	26	61
at least two consultations	17	39
Outpatient treatment during the first consultation (n = 43)			
yes	32	74
no	11	25
Laboratory tests during the first consultation (n = 43)			
yes	26	61
no	17	39
Main treatments received (n = 60)			
analgesics/antipyretics	41	68
antibiotics	36	60
antimalarials	14	23

Several factors were significantly associated with delayed consultation. The most decisive factors were unfavorable socioeconomic conditions and poor follow-up quality, both of which significantly increased the risk of delay. Using analgesics at home, self-medicating, and using traditional medicine also contributed to the delay in accessing care. Table IV summarizes the factors associated with delayed consultation.

**Table IV:** Distribution of patients according to factors associated with delayed consultation

	Delayed consultation		μOR [CI 95%]
	Yes (n = 43)	No (n = 17)	
Socioeconomic status			
high or moderate	6 (27 %)	16 (73 %)	0,0115 [0,0002; 0,0961]
low	37 (97 %)	1 (3 %)	
Pain medication at home			
yes	40 (83 %)	8 (17 %)	14,0534 [2,7646; 99,0895]
no	3 (25 %)	9 (75 %)	
Quality of follow-up			
good	8 (35 %)	15 (65 %)	0,0331 [0,0031; 0,1824]
poor	35 (95 %)	2 (5 %)	
Self-medication			
yes	30 (83 %)	6 (17 %)	4,1195 [1,1165; 16,8024]
no	13 (54 %)	11 (45 %)	
Traditional medicine			
yes	16 (94 %)	1 (6 %)	9,2174 [ 1,2012; 421,1291]
no	27 (63 %)	16 (37 %)	

## Discussion

For nearly three years, the pediatric department at Cocody University Hospital has maintained a follow-up cohort of children with SCD. The cohort includes patients who were diagnosed in outlying areas and referred to the department for follow-up, as well as children who were identified during hospitalization for complications and integrated into the cohort after treatment. The management of SCD patients in our department is still disorganized. Organizations fighting SCD primarily focus on raising awareness and providing support for patient education. However, parents are entirely responsible for all costs related to care, including medications, laboratory tests, hospitalization, and vaccinations.

The frequency of acute SCD complications among emergency department admissions was high. The high rate observed in our study could be explained by the closure of Yopougon University Hospital, which housed the clinical hematology department and was a referral center for the management of SCD. The closure led to patients being referred to the Cocody University Hospital.

The socioeconomic status of patients was predominantly low. This situation could hinder the proper follow-up of patients with SCD. A statistically significant association was found between socioeconomic conditions and time to consultation (p < 10^-3^). Patients from disadvantaged backgrounds sought care later, likely due to insufficient income limiting access to timely care. These results suggest that financial resources are a major determinant of the care pathway for patients with SCD.

A SCD diagnosis is often made when clinical manifestations occur, such as anemia, vaso-occlusive crises affecting the osteoarticular or abdominal regions in older children, and hand-foot syndrome in infants. This situation is frequently observed in countries with limited resources. For instance, Hernandez *et al*. reported a prevalence of 48% for vaso-occlusive crises and 25% for hand-foot syndrome in Uganda in 2018 [7]. This could be explained by the absence of neonatal screening for SCD. In our context, where systematic screening for SCD was rarely performed, complications thus constitute the circumstance of diagnosis. In contrast, systematic screening is performed during the neonatal period in Europe, specifically in France [3]. Late diagnosis also highlights the shortcomings of our healthcare system. When children experience recurrent anemia or abdominal pain, clinicians should order a hemoglobin electrophoresis as a first-line test, which is not routinely done.

Patient follow-up generally appeared inadequate. In our context, care for patients with SCD is not covered by a universal health insurance system. Although certain non-governmental organizations provide occasional support to a few families, this assistance is limited and does not cover the entire country. At the same time, patient associations for SCD play an essential role in improving the overall management of this chronic condition. Typically established as nonprofit organizations, these associations are often initiated and led by patients, their parents, or healthcare professionals. They operate on multiple levels, providing psychosocial support to families, offering therapeutic education to patients, and raising public awareness to promote early screening and reduce the stigma associated with the disease.

Additionally, these organizations advocate for greater recognition of SCD as a major public health issue with health authorities. They help organize screening campaigns, train patients and caregivers, and sometimes provide financial or material assistance to facilitate access to care, such as medications, additional tests, and hospitalizations.

However, despite their commitment, the impact of these associations is limited by several constraints: insufficient financial resources, dependence on external partners, a lack of organizational structure, and limited geographic coverage. Therefore, strengthening their institutional capacities and integrating them into national health programs appears essential for improving the quality of life and medical care of patients with SCD in the long term.

Families still largely bear the costs associated with laboratory tests, hospitalizations, and essential medications such as hydroxyurea or antibiotics. This socioeconomic reality undermines access to care, encourages self-medication and the use of traditional therapies, and may explain why many patients receive irregular medical follow-up. However, the use of traditional medicine cannot be explained solely by financial constraints. It is also rooted in sociocultural factors and healthcare-seeking behaviors, wherein families’ treatment choices are influenced by their beliefs. Another important finding of this study is the lack of a structured care pathway for children with SCD. Currently, there is no formalized care pathway, which undermines continuity and coordination of care. This disorganization leads to fragmented management with gaps in medical follow-up, irregular appointment attendance, and difficulty ensuring long-term treatment adherence. Establishing a referral center or specialized units dedicated to SCD would allow for the organization of coherent, patient-centered care pathways, improving both the prevention of complications and patients’ quality of life.

Since there is no curative treatment for SCD, a chronic condition, improving the prognosis requires regular follow-up to prevent life-threatening complications [8]. Our study revealed a significant association between the quality of medical follow-up and time to consultation (p < 10^-3^). Children who received irregular follow-up were more likely to delay seeking consultation. Conversely, regular, high-quality follow-up appeared to be a protective factor, facilitating earlier intervention. Literature data underscore the importance of structured, continuous follow-up in anticipating complications and ensuring rapid referral to healthcare facilities in the event of a crisis [5].

Vaccination coverage was often incomplete or poorly documented. This low vaccination coverage rate could be explained by ignorance, negligence, and a lack of awareness of the importance of properly vaccinating children with SCD. Vaccines outside of the EPI recommended by the World Health Organization for children with SCD are inaccessible in our setting due to cost [11]. These children should receive these vaccines free of charge because they are highly susceptible to infections caused by encapsulated bacteria, such as pneumococcus, meningococcus, *Haemophilus*, and salmonella. The lack of adequate vaccination coverage among children with SCD leads to serious infectious complications. This underscores the importance of strengthening vaccination strategies, improving therapeutic education for families, and promoting more rigorous medical follow-up.

In our study, a significant proportion of children had already sought medical care prior to admission, sometimes multiple times. This phenomenon can be explained by the fact that parents only seek care at the referral center when the initial treatment administered at the primary care level is ineffective. Olatunya *et al.* in Nigeria confirmed this trend, reporting a first-visit rate of 58.2% and highlighting the gradual implementation of the healthcare pyramid [9].

The most commonly prescribed drug classes were analgesics/antipyretics, antimalarials, and antibiotics. This trend is common in several African countries, including Nigeria [9]. This situation is likely related to the variety of complications observed in children with severe sickle cell syndrome.

In 71.7% of cases, patients sought medical care more than 24 hours after symptom onset.

Our study found that self-medication, use of traditional therapies, and administration of analgesics at home were significantly associated with delayed consultation (p = 0.023, p = 0.02, and p < 10^-3^, respectively). These intertwined practices can temporarily mask symptoms or create a perception of improvement, delaying the use of formal healthcare. This delay compromises the early management of complications and can worsen the prognosis. Targeted interventions combining improved financial and geographic access to care are needed, particularly through the establishment of community-based facilities and strengthening therapeutic education for families. This education must emphasize recognizing signs of severity early on and the limitations of self-medication. Integrating traditional healers into collaborative strategies could facilitate faster referrals to healthcare facilities.

This study has several limitations that should be noted.

First, the retrospective design makes it susceptible to incomplete data, especially regarding certain aspects of clinical follow-up and the parents’ occupations. Second, the small sample size limits the ability to conduct multivariate analyses. Additionally, the population receiving care at Cocody University Hospital is likely not representative of all children with SCD in Abidjan. Financial constraints may hinder access to this center, particularly for low-income families, resulting in underrepresentation in our sample. This selection bias must be considered when interpreting and generalizing the results. Finally, the lack of comprehensive data on the actual costs borne by families limits a detailed economic analysis of the care pathway.

## Conclusion

The assessment of the care pathway for children with SCD reveals shortcomings, including delays in seeking medical care driven by adverse socioeconomic conditions, self-medication, traditional therapy, and irregular follow-up, as well as the absence of a clear, structured care pathway. These limitations compromise the health and life prognosis of children, underscoring the urgency of appropriate interventions. Improving financial and geographic access to care, combined with enhanced therapeutic education for families, is an essential strategy for improving disease understanding, guiding self-medication, ensuring rigorous follow-up, and recognizing signs of severity early on. Furthermore, involving traditional healers within an approach that respects the sociocultural context should help reduce delays in seeking medical care. Lastly, establishing a specialized SCD referral center is crucial for structuring and coordinating care, ensuring continuity, and improving children’s prognosis in the long term.

In light of the identified limitations, multicenter, prospective studies on large samples should be conducted to improve the representativeness of the results. These studies should implement systematic data collection on costs associated with patient follow-up, including consultations, tests, treatments, and hospitalizations.

## Funding

No external funding was received for this study.

## Authors’ contributions

GRO-BI André Marius: Study design, data collection and analysis, and manuscript drafting.

MANSOU Komenan Amoro, DJIVOHESSOU Augustine, ASSI-SAHOUIN Ursule Anièla, Djoman Isabelle, KOUADIO Evelyne, DAINGUY Marie Evelyne, KOUAKOU Cyprien, AKE-ASSI Marie Hélène, N’GATTA Prisca, and SORHO Charlène: critical review, validation of results, and manuscript revision.

FOLQUET Amorissani: validation of the protocol and revision of the manuscript.

## Conflicts of interest

The authors declare no conflicts of interest

## Appendix: Individual survey form

Annexe : Fiche d’enquête
